# Food Insecurity, Dietary Intakes, and Eating Behaviors in a Convenience Sample of Toronto Youth

**DOI:** 10.3390/children9081119

**Published:** 2022-07-27

**Authors:** Alexandra Dubelt-Moroz, Marika Warner, Bryan Heal, Saman Khalesi, Jessica Wegener, Julia O. Totosy de Zepetnek, Jennifer J. Lee, Taylor Polecrone, Jasmin El-Sarraj, Emelie Holmgren, Nick Bellissimo

**Affiliations:** 1 School of Nutrition, Faculty of Community Services, Toronto Metropolitan University, 350 Victoria Street, Toronto, ON M5B 2K3, Canada; adubeltm@ryerson.ca (A.D.-M.); jwegener@ryerson.ca (J.W.); tpolecrone@ryerson.ca (T.P.); 2 Maple Leaf Sports and Entertainment LaunchPad, 259 Jarvis Street, Toronto, ON M5B 2C2, Canada; marika.warner@mlselaunchpad.org (M.W.); bryan.heal@mlselaunchpad.org (B.H.); 3 Physical Activity Research Group, Appleton Institute and School of Health, Medical and Applied Sciences, Central Queensland University, 160 Ann Street, Brisbane, QLD 4000, Australia; s.khalesi@cqu.edu.au; 4 Faculty of Kinesiology & Health Studies, University of Regina, 3737 Wascana Parkway, Regina, SK S4S 0A2, Canada; julia.totosy@uregina.ca; 5 Department of Nutritional Sciences, Temerty Faculty of Medicine, University of Toronto, 1 King’s College Circle, Toronto, ON M5S 1A8, Canada; jennjm.lee@mail.utoronto.ca; 6 Campus Aarhus N, VIA University College, Banegårdsgade 2, 8700 Horsens, Denmark; j.elsarraj@gmail.com (J.E.-S.); emelie.holmgren_94@live.se (E.H.)

**Keywords:** dietary intakes, eating behaviors, food insecurity, community programming, needs assessment

## Abstract

Background: Food insecurity has been shown to be associated with poor dietary quality and eating behaviors, which can have both short- and long-term adverse health outcomes in children. The objective was to investigate the food security status, dietary intakes, and eating behaviors in a convenience sample of youth participating in the Maple Leaf Sports Entertainment LaunchPad programming in downtown Toronto, Ontario. Methods: Youth aged 9–18 years were recruited to participate in the study. Food security status, dietary intakes, and eating behaviors were collected using parent- or self-reported questionnaires online. Results: Sixty-six youth (mean ± SD: 11.7 ± 1.9 years) participated in the study. The prevalence of household food insecurity was higher than the national average with at least one child under 18 years of age (27.7% vs. 16.2%). Dietary intake patterns were similar to the national trends with low intakes of fiber, inadequate intakes of calcium and vitamin D; and excess intakes of sodium, added sugar, and saturated fat. Despite a low prevalence of poor eating habits, distracted eating was the most frequently reported poor eating habit. Conclusions: Although youth were at high risk for experiencing household food insecurity, inadequate dietary intake patterns were similar to the national trends. Our findings can be used to develop future programming to facilitate healthy dietary behaviors appropriate for the target community.

## 1. Introduction

Approximately one in eight Canadian households experience food insecurity [1], which is defined as “the inability to acquire or access an adequate diet quality or a sufficient quantity because of financial constraints [2].” Individuals living in food insecure households are at higher risk of developing diet-related noncommunicable diseases, including obesity, type 2 diabetes, and cardiovascular diseases, and are more likely to report poorer mental health outcomes [3,4,5,6,7,8,9,10,11]. As financial instability is the most reliable indicator of food insecurity, the prevalence of food insecurity is typically higher among low-income households, households with children [1,2], and in areas with high living expenses, such as metropolitan areas [12]. For instance, according to the national survey, Canadian Community Health Survey 2017/18, 13.6% of households in Toronto reported experiencing food insecurity in the past year, which was higher than the national level of 12.7% [1].

Food insecurity has been associated with inadequate intakes of some nutrients and poor health outcomes. Canadian children aged 9–18 years in households experiencing food insecurity had a higher prevalence of inadequate intakes of some nutrients, including protein, vitamin A, and magnesium; and had higher intakes of energy-dense foods, which may influence weight status and have related long-term health consequences [10]. Further, children in food insecure households have been shown to have an increased likelihood of experiencing other physical (e.g., asthma), mental (e.g., anxiety and depression [8,9,11]), and social health consequences (e.g., trouble concentrating in school [7]).

As healthy eating is essential for children’s health, growth, and development, poor dietary intakes in childhood, whether in excess or inadequate, can lead to adverse health outcomes during childhood and increase the risk of developing chronic diseases later in life [13,14]. Yet, about one-third of Canadian children and adolescents report poor dietary intakes with excess energy intakes and inadequate vitamin and mineral intakes, particularly in fiber, calcium, and vitamin D [15]. In part, related to excess energy intake and subsequent weight gain, approximately 30% of Canadian children were overweight or obese as of 2017 [16], further corroborating the importance of healthy dietary habits among Canadian children and adolescents. Healthy eating also encompasses healthy eating habits and behaviors, which refer to “where, when, why, and how you eat [17].” Poor dietary habits (e.g., distracted eating, eating alone) have shown associations with markers of poor diet quality, including the consumption of energy-dense, low-nutrient foods and poor self-regulation of energy intake [18]. In children, eating habits and behaviors are particularly important, as they shape children’s experiences with food to influence short- and long-term dietary intakes [18]. 

Community-based programs have the potential to provide social support, help alleviate some of the burden related to food insecurity and improve dietary behaviors. Food-related community-based programs such as community kitchens have been shown to increase self-reliance and engagement with social services, improve social skills, and enhance social support [19,20]. Other community-based interventions have shown to help increase fruit and vegetable intakes and self-efficacy for making healthy food choices in children [21]. Evidence-based programming can help to develop effective initiatives and programs to target the specific needs of the community. However, food security and dietary data of specific communities are often limited, resulting in community centers relying on national or provincial data to inform programming, which may poorly represent the communities being served. Community centers, particularly those located in areas with risk factors associated with poor health outcomes (e.g., low income, communities with racial disparities) are in greater need of exploration in order to identify and address the specific needs of the likely underrepresented communities that they serve. Therefore, the objective of the present study was to examine the food security status, dietary intakes, and eating behaviors among youth attending a community center in a low-income neighborhood in a large metropolitan Canadian city (Toronto, Ontario) to guide program development and initiatives within a community center.

## 2. Materials and Methods

### 2.1. Study Design and Participants

A cross-sectional analysis of household food security status, dietary intakes, and eating behaviors in a convenience sample of youth in Toronto was conducted. Youth aged 9–18 years who were registered members of Maple Leaf Sports Entertainment LaunchPad (MLP) were recruited to participate. MLP is a not-for-profit community space for youth, located in Toronto, Ontario, which provides free Sport for Development programs, wraparound services, and nutritional supports for youth.

Recruitment took place in 2018–2019 via convenience sampling at MLP by trained research assistants during program registration, and before and after regular sports programming. Research assistants verbally explained the study and distributed study information to prospective participants. Additional recruitment took place by e-mailing individuals with MLP membership accounts and by posting recruitment advertisements within MLP. Interested participants and parent/guardian(s) provided written informed assent and consent, respectively. The study protocol was approved by the Ryerson University’s Research Ethics Board.

### 2.2. Data Collection

Data on demographics, food security status, dietary intakes, and eating behaviors were collected using questionnaires through MLP Scoreboard^TM^, an online interface where the MLP staff monitor and interact with registered youth members. Youth aged 9–17 years independently completed the dietary intake and eating behavior questionnaires, while their parent/guardian(s) provided demographic and food security status. Youth aged 18 years independently completed all four questionnaires.

The demographic questionnaire collected data on age, sex, household annual income, parental education, ethnicity, and birthplace.

Household food security status was assessed using a validated 18 item household food security survey module (HFSSM) [1,22,23]. The questionnaire asked about food security experiences, which ranged from access to adequate and appropriate food, to availability of food due to limited financial resources over the previous 12 months. The experiences ranged from worrying about running out of food, to the inability to afford a balanced diet, to missing meals, and to going a whole day without eating due to lack of food and money. The questionnaire assessed the food security status of adults as a group and children as a group within a household to capture the unique experiences of each group [1,22]. The questionnaire consisted of 10 questions pertaining to the household in general or adults in the household (i.e., adult scale), and 8 questions pertaining to children under 18 years old in the household (i.e., child scale). Food security status for the adult and child scale were categorized separately into 4 classifications (food secure; marginal food insecure; moderate food insecure; severe food insecure) based on the number of affirmative responses (i.e., food insecure experiences). Using the food security status categories for the adult and child scale, household food security status was categorized into one of the 4 classification systems: food secure; marginal food insecure; moderate food insecure; and severe food insecure [1,22].

Dietary intakes were assessed using a validated 177 item Harvard Youth/Adolescent Questionnaire (YAQ), a food frequency questionnaire that estimates the average nutrient intakes from foods, beverages, and supplements over the previous year in North American children and adolescents [24,25]. Self-reported height and weight were collected, then used to calculate the age- and sex-specific BMI percentile according to the Centers for Disease Control growth charts [26]. Completed YAQs were analyzed externally by the Harvard T.H. Chan School of Public Health [24,27]. YAQ data was excluded if the survey was incomplete (i.e., <80%), or if the total energy intakes were implausible (<500 or >5000 kcal/day) [24,25,28].

To assess the prevalence of nutrient adequacy, nutrient intakes were compared with the dietary reference intakes (DRIs) [29,30] or the recommended levels by dietary guidelines [31,32] when DRIs were not available. Macronutrient intakes (i.e., protein, carbohydrates, and total fat) were compared to the acceptable macronutrient distribution range (AMDR). Saturated fat and added sugar intakes were calculated as absolute values (g) and percentages of total energy, then compared with the recommended levels by the World Health Organization (WHO) (i.e., <10% of total energy) [31] and the U.S. Dietary Guidelines for Americans [32], respectively. Vitamin and mineral intakes were compared to the estimated average requirement (EAR) except for fiber, sodium, and potassium, as adequate intake (AI) levels were used due to the absence of EAR. Further, sodium intakes were compared with the Chronic Disease Risk Reduction Intake (CDRR) levels to assess the prevalence of excess sodium consumption [30].

Eating behaviors were assessed using a 20 item, 5 point Likert scale questionnaire related to eating habits, involvement in meal preparation, eating location, snacking occasion, and school day lunch habits. There were 8 questions adapted from the validated Family Activity and Eating Habits Questionnaire [33] and 12 questions related to youth’s involvement in meal preparation, snacking occasions, and other eating habits, which were identified as important behaviors related to children’s dietary intakes [34,35]. Each question was scored from 0 to 4 (0: never, 1: almost never, 2: sometimes, 3: frequently, 4: always).

### 2.3. Statistical Analyses

Descriptive statistics were computed for demographic characteristics, food security status, dietary intakes, and eating behaviors. Data are presented as means and standard deviations (SD) for continuous variables, frequency, and percentages for categorical variables; and as median and interquartile range (IQR) for Likert-scaled questions. The internal consistency reliability of the modified eating behavior questionnaire was measured using Cronbach’s α, with α levels ≥ 0.7 considered acceptable reliability [36]. All analyses were completed using SAS version 9.4 (SAS Institute Inc., Carey, NC, USA).

## 3. Results

Sixty-six youth (32 females, 34 males) with a mean ± SD age of 11.7 ± 1.9 years and BMI percentile of 61.9 ± 34.3 participated in the study and were included in the analyses. However, not all participants completed all the surveys: HFSSM was completed by 47 participants, YAQ by 63 participants, and the eating behavior questionnaire by 64 participants. The majority of the participants (88%) identified themselves to be racialized (i.e., non-Caucasian), with the highest level of representation reported being Black and Asian youth; and 36% of the participants reported an annual household income below CAD 30,000, which is below the national low-income cut-off (LICO) value of CAD 33,804 for four-person households [37]. Of the 47 participants who completed the HFSSM, 27.7% of the households were identified to have experienced food insecurity in the last 12 months. Food insecurity at the child level was lower than that of the adult level (17.0% vs. 27.7%). The internal consistency reliability showed an acceptable level of α = 0.740. Table 1 shows a detailed summary of the participant characteristics and food security status.

YAQ data from 3 participants was excluded due to implausible energy intake, resulting in the analysis of data from 60 participants. Estimated usual energy intake was 2319 ± 814 kcal/day with 95.1 ± 34.5 g/day of protein, 73.9 ± 30.8 g/day of total fat, and 332.5 ± 117.7 g/day of carbohydrate. Compared to the AMDR, 100%, 76.7%, and 91.7% of participants consumed within the recommended ranges for protein, total fat, and carbohydrates, respectively. Saturated fat intakes were 26.3 ± 12.5 g/day, contributing 10.1% of total energy intake with 50% of participants exceeding the recommended intake levels for saturated fat (i.e., 10% of total energy intake [31]). Total sugar intakes were 153.2 ± 62.1 g/day, contributing 26.5% of total energy intake. Added sugar intakes were 62.7 ± 33.3 g/day, contributing 10.7% of total energy intake, similar to the national added sugar intake levels of 10.3% among 2–18 year-old children [38], with 51.7% of participants exceeding the recommended level of 10% of energy intake [32]. Fiber intake was 28.4 ± 11.7 g/day, with 50% of participants consuming below the AI. A low prevalence of inadequate intakes (i.e., <30% below EAR) was observed for zinc, vitamin A, riboflavin, niacin, vitamin B_6_, folate, vitamin B_12_, and vitamin C. However, inadequate intakes in over 30% of the participants were observed for calcium, vitamin D, and vitamin E. Further, with an average sodium intake of 2979.1 ± 1264.5 mg/day, 81.7% of participants exceeded the CDRR. Table 2 shows a summary of the estimated energy and nutrient intakes and the intake levels compared to various DRI values.

Out of a maximum of 24 points, the median overall eating habits and style score was 7.5 [IQR: 4, 11], indicating a low prevalence of less favorable eating habits. The most frequent occurrence of less favorable eating habits was eating while watching TV, reading, or working, with a median score of 2 [2,3]. The median involvement in meal preparation scores were 2–3, with the most frequent preparation involvement reported for lunch. The median scores for eating in the living room, bedroom, and dining room were 2, 0, and 2.5, respectively, revealing the dining room to be the most frequently used eating location. A low frequency of snack consumption was reported (median ≤ 2). The majority of the participants reported regularly bringing school-day lunches from home (82.8%) as opposed to purchasing a lunch or returning home for lunch. Table 3 shows a summary of the responses to the eating behavior questionnaire.

## 4. Discussion

The present study examined household food security status, dietary intakes, and eating behaviors among youth attending a community center in a low-income neighborhood in a large metropolitan Canadian city (Toronto, Ontario). As community centers (including MLP) are hubs for facilitating the development of healthy behaviors and skills, it is important to understand the community’s dietary intakes, habits, and environments to optimize opportunities for health promotion. Our findings suggested a high prevalence of household food insecurity among the participants compared to the national household food insecurity rate. Similar to national dietary patterns, low intakes of fiber, inadequate intakes of calcium and vitamin D, and excess intakes of saturated fat, added sugars, and sodium were observed. The most frequent occurrence of poor eating habits was distracted eating.

Food insecurity indicates material deprivation; therefore, is heavily influenced by income. Concurrent with a high prevalence of households with low income (likely below the LICO), participants in the present study reported higher levels for all levels of household food insecurity compared to the national levels of households with children reported in 2017/18 [1]: marginal 8.5% vs. 5.1%, moderate 12.8% vs. 8.3%, and severe 6.4% vs. 2.9%. Of particular concern, more than double the national levels of severe food insecurity were reported. Severe household food insecurity signifies an extreme level of deprivation which compromises the quality and/or quantity of food intake among adults and/or children and has been strongly associated with adverse physical and mental health outcomes [3,4,5,6]. However, other social and economic factors including ethnicity, household characteristics (e.g., lone-parent vs. two-parent), and housing situation (e.g., rental vs. homeownership) [40] may contribute to the high prevalence of food insecurity observed in the study. Collaborations between community centers, government agencies, public health units, schools, and other nonprofit organizations are vital to address underlying systemic gaps of food insecurity while offering complementary services and resources for both youth and adults to achieve common public health goals for the community.

Although food insecurity may contribute to poor dietary quality and food choices [41], overall trends of nutrient intakes of the participants were similar to the national population of the same age groups [15]. Specifically, the high prevalence of low fiber, and inadequate calcium and vitamin D intakes were similar to observed nutrient intakes of children from the same age groups in Canada [15]. Adequate fiber intake in children and adolescents is associated with low risk of obesity, metabolic syndrome, insulin resistance, constipation, and hypertension [42]. Calcium and vitamin D are important nutrients for supporting bone health, growth, and development [43], particularly important during adolescence when over half of peak bone mass is acquired [44]. Contrary to the national intake levels, a high prevalence of inadequate vitamin E intake was observed. Vitamin E can help protect cells from oxidative stress and has been shown to improve cardiovascular and immune function [45,46,47,48]. Future studies examining intakes of different food groups can help identify the low consumption of specific foods (e.g., oils, nuts, and seeds for vitamin E [48]) that can be incorporated into programming. Further, sodium (2979 mg/day vs. 2350–3320 mg/day [49]), added sugar (10.7% vs. 10.3% of total energy [38]), and saturated fat (10.1% vs. ~10% of total energy [15]) intakes were similar to the reported intakes of the same age groups in Canada, all with a high prevalence of participants exceeding recommended levels that may increase the risk of adverse health outcomes, including hypertension, dental caries, and cardiometabolic outcomes [31,39,50]. Considering several inadequate and excess nutrient intakes identified in the current study, future research on dietary patterns is needed to complement the present nutrient-focused analysis and to help develop programming on both nutrient- and diet-oriented recommendations.

Despite the low prevalence of less favorable eating behaviors, distracted eating was most frequently reported with some involvement in meal preparation. Poor eating habits have been associated with poor diet quality and excess energy intakes [41,51,52]. In children, eating while watching TV has been associated with higher consumption of energy-dense, high-sugar, and high-fat foods; lower consumption of fruits and vegetables; high energy intake; and increased BMI z-scores [51]. Involvement in meal preparation has been shown to be associated with higher diet quality [34,35], and increased self-efficacy related to eating healthy foods (e.g., whole grains, fruits and vegetables) and developing cooking skills in children [53]. Parents/guardians play a significant role in shaping eating habits and behaviors in youth, as they regulate eating environments, provide early social interaction, and introduce healthy and culturally appropriate eating patterns [18,54]. Leveraging the parental role in eating behaviors in youth, family-based initiatives and programming can be helpful in targeting eating habits and creating healthy eating environments to build positive eating behaviors.

As part of the needs assessment, the findings of the study were used to make several recommendations to serve the community. The findings were used by MLP to develop targeted programming and initiatives, including: (1) creating a placement position to translate the study’s findings into practical recommendations for implementation in MLP programs; (2) informing a Nutrition Hub optimization plan in 2021 as part of MLP’s overall business operations; (3) initiating a process of regular, ongoing needs assessment for food insecurity and nutritional factors among MLP youth and families; and (4) implementing two programs to address priority recommendations identified in this study: The Family Food Program and the Healthy Me, Healthy Community Program. The Family Food Program focuses on family involvement and parent education on healthy eating by providing free and healthy food boxes of fresh produce, cooking instructions for meals and access to live cooking demonstrations and engagement opportunities with an MLP chef. The Healthy Me, Healthy Community Program is an education-based program to promote healthy eating, physical activity, and positive lifestyle habits. The program curriculum emphasizes a healthy diet based on whole foods, while reinforcing healthy eating patterns and reducing the reliance on highly processed foods in snacks and recipes.

In addition, the study findings suggest a need for a multilevel and/or systemic approach to achieve public health goals. For instance, more adults experience food insecurity than children, likely related to adults experiencing food deprivation to maximize food availability for their children [1,55]. Dietary intakes for adults in food insecure households may be compromised at a greater rate than that of children [1,55]. As a long-term solution to address the multifactorial causes of food insecurity and poor dietary intakes, dietitians and public health professionals can help develop partnerships and collaborations; plan, develop, and evaluate appropriate interventions; and lead advocacy work to narrow the systemic gaps for the community.

There are a few limitations to note. First, we had a small sample size, which may affect the generalizability of the findings. Despite having about 200 anticipated participants, the study participation was low, in part related to systemic barriers and inequities contributing to the underrepresentation of these vulnerable populations. Partnerships with other community groups and organizations are encouraged to improve recruitment of a larger sample with specific strategies to recruit underrepresented populations. Given the sample population and its size in the present study, our findings may not be generalizable to other populations; however, our study provides an important direction for future research and initiatives in the study population and highlights the need for other communities to undertake an evidence-based approach for targeted programming. Second, limited information was available to explore additional household characteristics, including the number of adults and children in households. It is unknown whether a participant lives in more than one household (and whether one or both households are food insecure) or if multiple participants live in the same household. Data collection on household composition, particularly combined with the HFSSM survey, could be beneficial for identifying the contribution of household characteristics to food security status. Lastly, we were limited in collecting directly measured anthropometrics and data on physical activity levels to estimate the energy requirements needed to maintain an energy balance in individuals [56]. Although parent-reported height and weight met the logistical and technical feasibility in the present study, they have been shown to be subject to reporting errors [57,58,59]. Physical activity is independently associated with physical and mental health benefits for youth [60,61]. Future studies directly measuring anthropometrics and assessing physical activity could be helpful in predicting energy needs and developing other lifestyle programming.

## 5. Conclusions

In conclusion, a convenience sample of youth in a Canadian metropolitan area at high risk for experiencing food insecurity showed similar inadequate dietary intake patterns as the national trends. Our findings suggest that food insecurity is not causally linked to poor dietary quality and eating behaviors, and evidence-based community/public health programs need to be combined with systemic approaches to address the root causes of food insecurity.

## Figures and Tables

**Table 1:** Participant Demographics.

Characteristics	
**Sex, n (%**)	
Female	32 (48.5)
Male	34 (51.5)
**Age, mean ± SD**	11.7 ± 1.9
BMI percentile *, mean ± SD	61.9 ± 34.3
BMI category *, n (%)	
Underweight (BMI percentile < 5th)	8 (13.3)
Healthy weight (5th ≤ BMI percentile < 85th)	32 (53.3)
Overweight (85th ≤ BMI percentile < 95th)	7 (11.7)
Obese (95th ≤ BMI percentile)	13 (21.7)
**Household annual income ^†^, n (%)**	
< CAD 20,000	8 (16.0)
~CAD 20,000–30,000	10 (20.0)
~CAD 30,000–83,000	3 (6.0)
> CAD 83,000	8 (16.0)
Unsure of annual income	21 (42.0)
**Highest level of education achieved by parent/guardian ^†^, n (%)**	
No high school diploma	8 (16.0)
High school diploma or equivalent	5 (10.0)
Some college credit, no degree	13 (26.0)
Trade/technical/vocational training	4 (8.0)
Bachelor’s degree	9 (18.0)
Postgraduate degree	11 (22.0)
**Ethnicity ^†^, n (%)**	
Caucasian	6 (12.0)
Black	13 (26.0)
Asian	15 (30.0)
Other	16 (32.0)
**Birthplace ^†^, n (%)**	
Canada	40 (80.0)
Outside of Canada	10 (20.0)
**HFSSM ^‡^—Household Food Security, n (%)**	
Food secure	34 (72.3)
Marginal food insecurity	4 (8.5)
Moderate food insecurity	6 (12.8)
Severe food insecurity	3 (6.4)
**HFSSM ^‡^—Adult Food Security, n (%)**	
Food secure	34 (72.3)
Marginal food insecurity	4 (8.5)
Moderate food insecurity	6 (12.8)
Severe food insecurity	3 (6.4)
**HFSSM ^‡^—Children Food Security, n (%)**	
Food secure	39 (83.0)
Marginal food insecurity	4 (8.5)
Moderate food insecurity	2 (4.3)
Severe food insecurity	2 (4.3)

*n* = 66. Abbreviations: BMI, body mass index; HFSSM, household food security survey module. * 60 participants provided self-reported height and weight, which was used to calculate age- and sex-specific BMI percentiles according to the Center for Disease Control growth charts [26]. ^†^ 50 participants provided additional demographic information. ^‡^ 47 participants completed the HFSSM.

**Table 2:** Usual energy and nutrient intakes estimated using the Youth and Adolescent Questionnaire (YAQ).

Nutrients	Mean ± SD	Proportion to Energy Intake (%), Mean ± SD	< EAR or AI *, n (%)	> CDRR or Dietary Guidelines ^†^, n (%)
Energy (kcal/day)	2319 ± 814			
Protein (g/day)	95.1 ± 34.5	16.6 ± 2.4		
Total fat (g/day)	73.9 ± 30.8	28.1 ± 4.5		
Saturated fat ^†^ (g/day)	26.3 ± 12.5	10.1 ± 2.1		30 (50.0)
Trans fat (g/day)	1.3 ± 0.7			
Carbohydrate (g/day)	332.5 ± 117.7	57.9 ± 6.3		
Total sugars (g/day)	153.2 ± 62.1	26.5 ± 5.7		
Added sugars ^†^ (g/day)	62.7 ± 33.3	10.7 ± 4.7		31 (51.7)
Fiber * (g/day)	28.4 ± 11.7		30 (50.0)	
Calcium (mg/day)	1399.8 ± 674.0		18 (30.0)	
Iron (mg/day)	20.1 ± 10.2		0	
Sodium * (mg/day)	2979.1 ± 1264.5		5 (8.3)	49 (81.7)
Potassium * (mg/day)	3554.8 ± 1315.1		11 (18.3)	
Phosphorous (mg/day)	1687.1 ± 628.7		10 (16.7)	
Magnesium (mg/day)	380 ± 134.8		9 (15.0)	
Zinc (mg/day)	15.4 ± 7.6		4 (6.7)	
Vitamin A (µg RAE/day)	1319.4 ± 714.2		2 (3.3)	
Thiamin (mg/day)	2.2 ± 1.0		0	
Riboflavin (mg/day)	2.8 ± 1.3		1 (1.7)	
Niacin (mg/day)	30.3 ± 14.1		0	
Vitamin B_6_ (mg/day)	2.8 ± 1.3		0	
Folate (µg/day)	624.2 ± 285.2		3 (5.0)	
Vitamin B_12_ (µg/day)	7.3 ± 4.7		2 (3.3)	
Vitamin C (mg/day)	200.2 ± 123.1		2 (3.3)	
Vitamin D (IU/day)	572.4 ± 372.5		21 (35.0)	
Vitamin E (mg/day)	18.0 ± 20.6		20 (33.3)	

Usual energy and nutrient intakes were estimated by using self-reported Youth and Adolescent Questionnaire (YAQ); *n* = 60. Intake adequacy was assessed by comparing the estimated intake levels with various dietary reference intake (DRI) values (i.e., AMDR for macronutrients, EAR or AI for vitamins and minerals, and CDRR for sodium) of corresponding age-sex DRI groups [29,30]. * In the absence of EAR, AI was used to compare the estimated intake levels. ^†^ Due to the absence of DRI values, the WHO recommendations for all populations were used [31,32,39]. Abbreviations: AI, adequate intake; AMDR, acceptable macronutrient distribution range; CDRR, chronic disease risk reduction intake; EAR, estimated average requirement; RAE, retinol activity equivalents.

**Table 3:** Summary of the eating behavior questionnaire.

Eating Behavior Questions	
**Eating habits, median [IQR]**	
Eat while standing	1 [0,2]
Eat straight from the pot/baking pan/frying pan	0 [0,1]
Eat while watching TV, reading, working	2 [2,3]
Eat when bored	1 [0,2]
Eat when angry or in other negative mood states	0.5 [0,2]
Eat late in the evening or at night	1 [1,2]
Overall eating habit score *	7.5 [4,11]
**Involvement in meal preparation, median [IQR]**	
Breakfast	2 [2,3]
Lunch	3 [2,4]
Dinner	2.5 [2,4]
Snacks	2 [2,3.5]
**Eating location, median [IQR]**	
Living room/TV room	2 [2,3.5]
Bedroom	0 [0,1]
Dining room	2.5 [0,4]
**Snacking occasion, median [IQR]**	
Between breakfast and lunch	2 [2,3]
Between lunch and dinner	1 [2,3]
After dinner	1 [1,2]
**School day lunch, n (%)**	
Returning home for lunch	3 (4.7)
Buying at school	6 (9.4)
Prepared lunch from home	53 (82.8)
Other	2 (3.1)

All eating behavior questions were conducted on a five-point Likert scale from 0 (“Never”) to 4 (“Always”); *n* = 64. * Overall scores were calculated by summing up the scores from 6 eating habit questions; higher score indicates less favorable eating behaviors. Abbreviations: IQR, interquartile range.

## Data Availability

The data presented in this study are available on request from the corresponding author.
